# Miscellaneous Notes

**Published:** 1875-08

**Authors:** 


THE *Chicago Medical Examiner* and the *Chicago Medical Journal* have been purchased by an incorporated stock company, composed of leading medical men of Chicago, and styled the "Chicago Medical Press Association." The two Journals are to be consolidated and issued monthly as the *Chicago Medical Journal and Examiner.* W. B. Keen, Cooke & Co. are the publishers, and the subscription price is fixed at four dollars per annum.

---

SPEEDY DEATH FROM SNAKE BITE.—In the same paper we find also the following: "A daughter, about twelve years old, of Mr. C. B. Culverson, who lives a few miles from this city, was out Friday, picking blackberries on the roadside near her father's residence. A gentleman who was riding by at the time, hearing her scream, hastened to her, and found that she had been bitten by a snake. The gentlemen quickly took out his knife and cut the flesh entirely out of the place where she had been bitten, but the poison had done its work, and in less than fifteen minutes the child was dead.

---

AN INTERESTING LECTURE by Dr. Brown-Sequard, on some new views concerning the localization of the functions of the brain, will appear in an early number of the *Boston Medical and Surgical Journal.* Other important articles are announced, including one on the treatment of typhoid fever by cold water, by Prof. R. T. Edes, and one on the non-restraint method adopted for the treatment of the insane at Dr. Fraser's asylum in England. Prof. Henry J. Bigelow will shortly publish an interesting paper on exstrophy of the bladder; and Prof. R. H. Fitz will contribute some original investigations on the pathology of nymphoangeioma.

---

ZIEMSSEN'S CYCLOPÆDIA OF MEDICINE.—On page 290 of Vol. III, second text line from the bottom, the word "ounces" should read "drachms." As the error might lead to serious consequences, subscribers to the work would do well to turn at once to the volume and correct this error.

---

"THE NATIONAL EXPOSITION of Agriculture, Horticulture, Mechanics, Minerals and Art" will open at Rome, Georgia, October 4th, and continue to October 9th, 1875. Half-rates have been obtained on all the leading railroad lines.

